# The Book World of Medicine and Science

**Published:** 1907-03-23

**Authors:** 


### The Book World of Medicine and Science.

Folia Therapeutica. A Journal of Modern Therapeutics and Pharmacology. Edited by J. Snowman, M.D., M.R.C.P. (London: Bale, Sons, and Danielsson, Limited. Published quarterly. Price 1s.)

As is suggested by its somewhat pedantic title, this new journal proposes to devote itself to the interests of therapeutics. It sketches an ambitious programme, the development of which will doubtless be watched with all goodwill. Nevertheless, the editor has a delicate task in hand. Of all departments of medical practice treatment most readily lends itself to irresponsible utterances, and there is ever danger lest the commercial schemes of the manufacturing druggist should manage to secure a foothold in pages professedly devoted to scientific discussion. Journals of such a complexion are not unknown, and we have no desire to see their numbers increase. If this journal can be kept free from all such influences we may expect the new venture to have a useful career in front of it. Possibly none of the articles can claim a very original quality, but they are all of practical value. The abstracts from articles bearing on therapeutics are well presented, and there is a useful list of current literature. We should like to have seen the guarantee of an editorial committee of recognised authorities, for only in this way, so it seems to us, can security be obtained for the sustained quality, independence, and worthy character of a journal devoted to treatment.

